# Temperature controlled microcapsule loaded with *Perilla* essential oil and its application in preservation of peaches

**DOI:** 10.3389/fnut.2023.1087605

**Published:** 2023-02-06

**Authors:** Zhigang Tai, Minjie Zheng, Ye Yang, Cheng Xie, Zhenjie Li, Chunping Xu

**Affiliations:** ^1^Faculty of Life Science and Technology, Kunming University of Science and Technology, Kunming, China; ^2^Yunnan Key Laboratory of Tobacco Chemistry, R&D Center of China Tobacco Yunnan Industry Co., Ltd., Kunming, China; ^3^College of Food and Bioengineering, Zhengzhou University of Light Industry, Zhengzhou, China

**Keywords:** *Perilla* essential oil, microcapsule, thermal property, preservation, peach

## Abstract

In this study, *Perilla frutescens* essential oil (PEO) loaded microcapsules (PEOM) were successfully prepared and their thermal stability, temperature-responsive releasing effect, antioxidant activity, antibacterial activity, and preservation of peach were systematically investigated. PEOM showed excellent encapsulation efficiency (91.5%) with a core-shell ratio of 1.4:1 and exhibited high thermal stability, indicating that PEOM could effectively maintain PEO release rate. *In vitro* assays indicated that the optimal kinetic model for PEO release fitted well with first order with a diffusion mechanism. A high level of antioxidant and antibacterial activity of PEOM was maintained. In addition, owing to its sustained release, PEOM could prolong the shelf life of peaches significantly. Therefore, PEOM has potential application and development prospects in the field of food preservation.

## 1. Introduction

Implied by the common saying “hunger breeds discontent,” living quality is strongly associated with food. Nowadays, in a rapidly developing global economy, transportation of fresh food over any great distance is an issue. Preservation methods are an important consideration to avoid contamination with pathogenic microorganisms and the rotting of food during storage (1). While chemical preservation agents are widely applied in food preservation, they can be detrimental to health and the environment and their use may be forbidden in the future (2).

As safe and environmentally more benign natural resources, plant essential oils are finding increasing application in food preservation (3). *Perilla frutescens* (L.) Britton (*Perilla*) is extensively cultivated in China as a spice and medicine, with health-supporting actions such as antioxidant, antisepsis, and detoxification (4). *Perilla* essential oil (PEO) extracted from *P. frutescens* is widely used for keeping food fresh (5). The Bai, Hani, and Dai tribes of the Yunnan province use PEO as a preservative, eliminating “fire toxin” accumulated in food (6). PEO contains fatty acids, monoterpenes, and sesquiterpenes, among other active components possessing a broad spectrum of biological activity, such as antioxidant and antibacterial (7). For example, the chemical constituents, antioxidants, and antimicrobial compounds of PEO were investigated by Chen et al. (8), finding higher DPPH than ABTS radical scavenging activity, comparable to the excellent antimicrobial activity of *Aspergillus niger* and *Candida albicans*. In, Chen et al. (9) found that PEO extracted by microwave-assisted hydrodistillation showed good antioxidant activity with an IC_50_ of 2.60 mu/ml, and excellent activity against A549 lung cancer cells and MGC803 human gastric cancer cells in comparison with paclitaxel. Recently, the mechanism of antibacterial activity of PEO toward *Aspergillus flavorosa* was observed by changes in phenotypic results (10). However, although PEO exhibits excellent biological activity and could be an effective antimicrobial agent with potential applications for food, drug, and health products, its instability to factors such as oxidation, pH, enzymes, and temperature, causes undesired variability in its chemical integrity, limiting its efficacy in these applications.

Various methods have been proposed and developed to improve the stability of essential oils and control their volatility (11). An effective method involves preparation of a chemical precursor involving a chemical bond between a matrix and the unstable component which could be released upon application of temperature, pH, or an enzyme (12). However, this approach is not suitable for component mixtures (13). For complex mixtures such as essential oils, the embedding approach is effective, introducing a shell as a diffusion barrier, enhancing the stability, and controlling the release and retention (14). Recent literature reveals that essential oil-loaded microcapsules have potential in food preservation, with excellent properties of effectiveness and low loss. For example, Sun et al. (15) found that fennel essential oil-loaded microcapsules effectively extended the life of pork, reducing the aerobic bacterial count and delaying the perishing of protein. Shao et al. (16) developed a packaging paper sprayed with cinnamon essential oil-loaded microcapsules, effective for extending the preservation of edible fungi.

Recently, various efficient encapsulation methods have been proposed, including emulsification (17), spray drying (18), microfluidic encapsulation (19), complex coacervation (20), and simple coacervation (21). In particular, the spray drying method has attracted much attention for its high yield, speed, and ease. However, the drying temperature is higher than in other methods, precluding its application to unstable essential oils (22). The coacervation method is attractive for essential oils, with room temperature operation and high productivity (23). For example, Chen et al. (24) reported that PEO microcapsules encapsulated by complex coacervation could increase the stability of PEO and were employed in food preservation. The process of complex coacervation is often induced by electrostatic attraction between polymers and surfactants, so several factors such as surfactant structure, the ratio of polymers, pH, charge characteristics, hydrophobic properties, and temperature should be considered carefully (20, 25). However, the method of simple coacervation proceeds by solubilization and coacervation of polymers and biomacromolecules and is conveniently modified by ion strength. Applied to microcapsules, the method is characterized by easy encapsulation, relatively rapid application, simple operation, and lower costs (21, 26). Therefore, simple coacervation is a more effective means to prepare PEO-loaded microcapsules, potentially improving stability, reducing PEO loss, improving the controlled release profile and widening the scope of application. Analysis of the literature revealed that *Perilla* essential oil microcapsules (PEOM) prepared by simple coacervation has not been investigated systematically or in depth.

Therefore, this study aimed to develop PEOM by simple coacervation and investigate their application in food preservation. The surface morphology, structure, and thermal properties of PEOM were investigated employing an inverted microscope (IM), a scanning electron microscope (SEM), Fourier Transform Infrared Spectroscopy (FTIR), X-Ray Diffraction (XRD), thermogravimetric analysis/differential scanning calorimetry (TG/DSC), and off-line thermolysis coupled with gas chromatography-mass spectrometry (OLT/GC/MS). The *in vitro* release antioxidant and antibacterial activities of PEOM were also evaluated. The application of PEOM to peaches was also investigated with very promising results, not only as a basis for further study on the preparation and thermal properties of PEOM but also demonstrating their potential as a food preservative.

## 2. Materials and methods

### 2.1. Materials

Gelatin (GE) (12.5%) was produced by Shandong Dakai Biotechnology Co., Ltd. (Shandong, China). PEO was purchased from Huaxin plant essential oil company (Yunnan, China). DPPH, TPTZ, transglutaminase (100 U/g of activity), Potassium iodide, chloroform, sodium thiosulfate, starch, n-hexane, petroleum ether, DMSO, sodium acetate, acetic acid, sodium hydroxide, ferric chloride, and anhydrous sodium sulfate were all analyzable pure reagents, which purchased from Sinopac Group Co., Ltd. (Beijing, China). *Bacillus subtilis*, *Staphylococcus aureus* and *Escherichia coli* were purchase from Agricultural Culture Collection of China (ACCC). Peach (Amygdalus persica) was purchased from the plantations (102°0.80′E, 24°0.89′N, Kunming city, China) in august 2022.

### 2.2. Preparation of POEM

The preparation of POEM by simple coacervation was accorded to the report by Gu et al. (27). In brief, 200 ml of GE solution (1 wt%) was stirred at 60°C until dissolved. 11.290 mg of PEO was added and emulsified by a high-speed agitator (Kexing, Shanghai, China) at 15,000 rpm for 8 min to obtain oil/water emulsion, followed by adjusting the pH value at 3.7 with acetic acid (10%, v/v). Sodium sulfate solution (20 wt%) was added dropwise within 3 min, then the amount of sodium sulfate dilution (10 wt%) was put into, and the mixture was stirred continuously at 0°C for 2 h. Encystation, dispersity, and coacervation of formed microcapsules were observed by using an IM (DM500, Lycra, Germany). Furthermore, a solution of enzyme transglutaminase (100 U/g of activity) was added to solidify the envelope. After stirring at 600 rpm for 1 h, suspension liquid was collected by filtration and water scrubbed three times. A freeze-drying process at −45°C for 72 h was carried out. Finally, the obtained PEOM has sealed preservation at 4°C in case of need.

### 2.3. Characterization analysis of PEOM

*Perilla* essential oil microcapsules surface was investigated by SEM (Quanta200, FEI, USA). The Structure of PEOM was measured by FTIR spectrometer (Nicolet iS20, Thermo Scientific, USA) within the scope of 400–4000 cm^–1^. Briefly, 8.0 mg of PEO, PEOM, and GE were mixed with potassium bromide and ground, respectively. Then, the powders were tableted and detected in the range of 400–4,000 cm^–1^ with an increment of 4 cm^–1^. In addition, the microscopic structure of PEOM was analyzed by XRD (Malvern, Egham, United Kingdom).

### 2.4. Encapsulation efficiency analysis

The encapsulation efficiency was investigated through the release of PEOM by using the method of extraction (28). 300 mg of PEOM was put into the 40 ml of water, followed by stirring at speed of 800 rpm. After 8 min, the PEOM was broken by ultrasound at 80% for 10 min. To extract PEO, 15 ml of petroleum ether was added to the mixture, blended well for 5 min, and stand for 30 min. The petroleum ether extract phase was separated from the mixture by centrifugal forces at 5,000 rpm for 8 min. Finally, the content of the petroleum ether extract phase was analyzed at 300 nm by using an Ultraviolet-visible spectrophotometer (UV-1780, Shimadzu, Japan). PEO concentration vs. absorbance of the calibration curve was shown in Supplementary Figure 2, and the equation of the standard curve was *Y* = 2.8323 X-0.0110, with a linear relationship of *R*^2^ = 0.9965. The encapsulation efficiency of PEOM was calculated by the following equation:

Encapsulationefficiency(EE%)=(W/W)0×100

Where W is the content of PO in microcapsules (mg), W_0_ is the initial PO amount (mg).

### 2.5. Thermal property of PEOM

#### 2.5.1. Thermal stability of PEOM

The thermal stability of PEOM was evaluated by using TG and DSC analysis (STA8000, PerkinElmer, USA). Before examination, the temperature was held at 800°C for 15 min to remove the impurity. 15 mg PEOM was put into a crucible. The temperature was in the range of 30.5°C−800°C at a heating rate of 10°C/min in the nitrogen flow rate of 10 ml/min.

#### 2.5.2. Thermolysis of PEOM

Thermolysis of PEOM was carried out as the description by our group with some modifications (29, 30). 500 mg of PEOM was put into an offline pyrolysis tank (OLT) self-made by our research group. And then, the OLT was sealed and placed in an automatic temperature-controlled electric furnace (Furnace 1,000°C, Tianjin, China) (31). The furnace temperature reached 100°C, and nitrogen as the carrier gas passed continuously through OLT for 10 min, meanwhile, decomposition products blown by carrier gas were absorbed by the mixture (hexane: methylene chloride: ethyl acetate = 1:1:1). Thermal decomposition products were detected by using the method of GC/MS (Agilent, Palo Alto, CA, USA). DB-5MS quartz capillary column (30 m × 0.25 mm × 0.25 μm, Agilent, USA) was used for the GC/MS analysis. Helium (99.999%) was used as carrier gas; the injector was at 250°C and the gas flow rate was set to 1.0 ml/min with splitting mode. Instrument conditions were as follows: initial temperature was set at 50°C for 3 min, then followed with a rate of 7°C/min up to 280°C, and kept for 10 min. The ion source of MS was set from 45 to 500 (m/z) with electron ionization of 70 eV. Temperature of ion source and quadrupole were 230°C and 150°C, respectively. Mass spectrometric data of constituents was retrieved from MS databases of NIST14, peak area percentage was employed to proceed with quantitative analysis.

### 2.6. *In vitro* release of PEOM

*In vitro* release of PEOM was investigated by using the methods described by Chen et al. (28) with some modifications. 150 mg of PEOM was placed in constant temperature equipment at 15°C, 25°C, 35°C, and 45°C. At intervals, the modest PEOM was taken out and dispersed into 30 ml of deionized water at 35°C. After stirring for 20 min, ultrasonic-assisted extraction was carried out at 35°C for 3 min, followed by extracting with petroleum ether at atmosphere temperature. The petroleum ether phase was separated by centrifugation for 10 min at 5,000 rpm. The PEO concentration was detected at 300 nm by UV. Finally, the cumulative release of PEO could be calculated as the following equation:

CumulativeRelease(CR%)=(M/M)0×100

Where M is the amount of PEO released from PEOM, and M_0_ is the initial amount of PEO in PEOM.

### 2.7. Antioxidant and antibacterial activity assay

#### 2.7.1. Sample pretreatment

Sample pretreatment of PEOM was carried out as described by Wang et al. (32). Briefly, antioxidant capacity and antibacterial activity were measured every 1 day, during 5 days. Before the first test, a suitable amount of the PEOM was put into the 5 ml of DMSO solution containing PEO of 5 mg/ml, followed by destruction assisted by ultrasound at 80% for 5 min, which should be guaranteed full release. In the following experiment, the wall quantity should be the same as the first test to ensure comparability in the follow-up experiments. The antioxidant and antibacterial activity were analyzed by using a DMSO supernatant.

#### 2.7.2. DPPH scavenging capacity

The DPPH scavenging capability was investigated as the paper described by Tai et al. (33) with some modifications. 100 μl of sample solution was added to 4.0 ml DPPH solution (0.1 mM). After shaking slightly, the reaction solution was kept in dark for 30 min. Then, the absorbance of reaction solution was measured at 515 nm by a UV spectrophotometer. In addition, 100 μl of ethyl alcohol with 4.0 ml DPPH solution (0.1 mM) served as blank control. DPPH scavenging capacity was calculated by using the following formula.

DPPH⁢scavenging⁢capacity⁢(DSC%)= [(A-blank⁢controlA)sample/A]blankcontrol×100%

Where A_blank control_ and A_sample_ are absorbancy of blank control and sample, respectively.

#### 2.7.3. Ferric-reducing antioxidant power (FRAP) reducing capacity

Ferric-reducing antioxidant power-reducing capacity was evaluated by the method described by Tai et al. (33). Briefly, a mixture of 5 ml of FeCl_3_ (20 mM), 5 ml of TPTZ (10 mM), and 50 ml of sodium acetate buffer (pH 3.5, 300 mM) as FRAP solution was freshly prepared. 100 μl sample, 3 ml of FRAP solution, and 300 μl deionized water were mixed and incubated at 37°C. After 45 min, the mixture was taken out, cooled at room temperature, and absorbance was recorded at 595 nm. Different concentration of ferrous ion solution was employed to draw a standard curve. All reagents were prepared on the same day.

#### 2.7.4. Antibacterial activity assay

Antibaterial activity was measured as the method of AGAR disk diffusion described by Silva et al. (34). The Sample solution was sterilized by UV for 40 min. Process of dilution and inoculation was performed on the benchtop. *S. aureus*, *E. coli*, and *B. subtilis* were diluted to obtain suspension with a concentration of 106 CFU/mL. Agar in a petri dish was punched into four wells with a diameter of 5.5 mm, and then the solution of PEO, PEOM, and GE were placed into each well. To avoid the loss of the sample, a drop of agar medium was added to seal the sample solution. Then, the petri dish was put in an incubator for 24 h at 37°C. The antibacterial activity was evaluated by gauging the diameter of bacterial inhibition ring. A petri dish without any sample was used as blank control and ampicillin was served as positive control group.

### 2.8. Preservation assay

The ability of preservation was investigated according to the report by Ban et al. (35). Fresh peach was randomly divided into control and treatment groups and kept in sealed plastic bags at room temperature for 10 days. PEOM enveloped in a cloth bag was pre-putted into plastic bags with the treatment group. Assessment of peach quality was performed from the following aspects: change appearance, hardness, titratable acidity (TA), soluble solid content (SSC), and total bacterial counts (TBC). The change in the appearance of peach was observed by naked eye, and taken a photo to capture changes. Hardens was evaluated by a fruit hardness tester (Bareiss, Berlin, Germany). Further, the most frequently technique, such as cold storage preservation (10 days, 4°C) was carried out to compare the propose method.

Freshness parameters including SSC and TA were measured as described by Qiang et al. (36). 100 g of Peach was ground for 10 min, following filtered by gauze, and a drop of juice was applied to the refractometer (Rudolph, New York, NY, USA). Results of SSC were recorded with corrected temperature. On the other hand, 100 g of peach was milled to measure the TA. The resulting homogenate was collected and heated at 55°C for 1 h. After cooling to 25°C, 5.000 g of homogenate sample was diluted with some water and titrated with a standard solution of sodium hydroxide with phenolphthalein indicator. TA was calculated by an equation:

TA⁢(mmol/100⁢g)=(C×V)/5×100%

Where C is the concentration of a standard solution of sodium hydroxide and V is the consumed volume.

Total bacterial counts of peaches were measured. In Brief, 10 g of peach was homogenized with 90 ml of sterile water for 30 min under aseptic conditions. Then, the as-prepared samples were coated on plate count agar and incubated at 37°C for 48 h. TBC was calculated by the following equation:

TBC=[N/(10×(V/90)]×100%

Where N is the count of bacteria. V is the volume of as-prepared samples.

### 2.9. Statistical analysis

Each experiment was performed three times for each sample. The result was analyzed by using the software of Origin 8.0.

## 3. Results and discussion

### 3.1. PEOM Characteristics

#### 3.1.1. PEOM morphology

**FIGURE 1:**
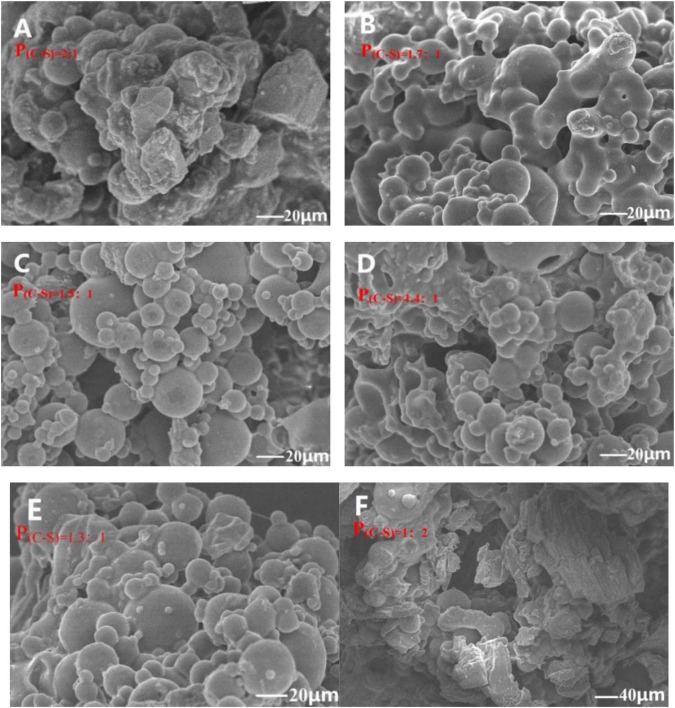
Microstructure of *Perilla* essential oil microcapsules (PEOM) with different core-shell ratio [P_(C–S)=2:1_ **(A)**, P_(C–S)=1.7:1_ **(B)**, P_(C–S)=1.5:1_ **(C)**, P_(C–S)=1.4:1_ **(D)**, P_(C–S)=1.3:1_ **(E)**, P_(C–S)=1:2_ **(F)**, Supplementary Figure 3] [For example, P_(C–S)=2:1_ indicated core-shell ratio was 2:1].

The properties of microcapsules are related to its morphology (37). Therefore, an IM and SEM were used to character the morphology of microcapsules. During the preparation process, the solubility of the wall material in the oil/water emulsion system is decreased by gradually adding anhydrous sodium sulfate, resulting in coacervation, and encapsulation of the PEO droplet. Microcapsules showed a hollow microsphere and good dispersion in water in the IM image in Supplementary Figure 1, indicating that PEOM was successfully prepared by simple coacervation. As shown in Figures 1A–F, various PEOM morphologies were observed when different core-shell proportions [P_(C–S)_] were applied. The PEOM were clearly spherical with a size of 30–40 μm (Supplementary Figure 3), with a smooth surface and no apparent cracks. The encapsulation efficiency (EE%) of PEOM with different core-shell ratios [P_(C–S)_] had an order of 91.5% [P_(C–S)=1_._4:1_] > 89.3% [P_(C–S)=1_._5:1_] > 86.8% [P_(C–S)=1_._3:1_] > 68.5% [‘P_(C–S)=1_._7:1_] > 30.5% [P_(C–S)=2:1_] > 21.3% [P_(C–S)=1:2_]. PEOM surface smoothness with various P_(C–S)_ was found to be, in sequence, P_(C–S)=1_._4:1_, P_(C–S)=1_._5:1_, P_(C–S)=1_._3:1_, P_(C–S)=1_._7:1_, P_(C–S)=2:1_, and P_(C–S)=1:2_. These results indicated that optimal encapsulation efficiency and surface smoothness were in the core-shell ratio range of 1.3:1-1.5:1. Regarding EE%, the core-shell ratio plays an important role. In this work, increasing the core-shell ratio from 1.3:1 to 1.5:1, the EE% reached a maximum of 91.5% at core-shell ratio 1.4:1 and then decreased. Furthermore, PEOM prepared with a core-shell ratio of 2:1 and 1:2 had low EE% of 30.5% and 21.3%, respectively. A large excess of either PEO or the shell material impedes efficient encapsulation (38). In addition, some small bumps on the surface of PEOM were found, perhaps due to extrusion during the freeze-drying process (39). Compared to tea tree oil microcapsules prepared by simple coacervation, PEOM obtained in this study showed higher EE% (40). In conclusion, PEOM prepared by simple coacervation with a core-shell ratio of 1.4:1 has a smooth surface and excellent encapsulation efficiency.

#### 3.1.2. FTIR and XRD analysis

Efficient binding between PEO and GE is crucial to ensure the stability and practical application of PEOM. FTIR and XRD were used to analyze the interaction between PEO and GE. The functional groups of PEO, GE, and PEOM were analyzed by FTIR to determine whether PEO was encapsulated by comparing peak shape, position, and intensity changes in the range of 400–4,000 cm^–1^ **(41)**. As shown in GE, a broad peak at 3,434 cm^–1^ was associated with the −OH stretching vibration. Another vibrational signal at 1,352 cm^–1^ is associated with the C-N group. Stretching vibrations associated with the carboxy group are evident at 1,605 and 1,460 cm^–1^. These results are consistent with the report by Hashim et al. **(42)**.

**FIGURE 2:**
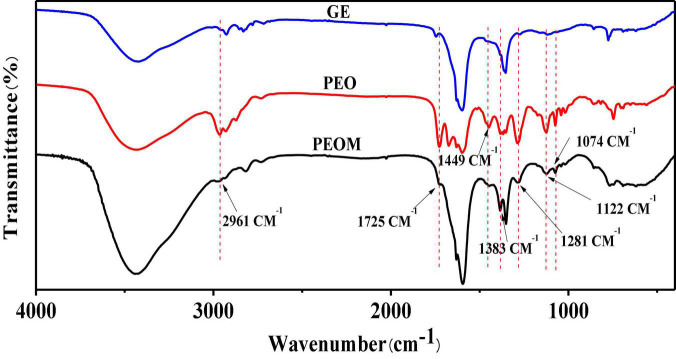
Fourier Transform Infrared Spectroscopy (FTIR) spectroscopy of *Perilla* essential oil microcapsules (PEOM).

The FTIR spectrum of PEO is also shown in Figure 2. PEO bands are associated with −OH stretching, −C = O vibration, methylene and C = C-H groups at 3,431, 1,592, 2,961, and 2,930 cm^–1^, respectively. Other characteristic signals of PEO associated with C-C-H groups, CH_2_ bending, O-C band, and −CH = CH- C bending are evident at 2,867, 1,383, 1,074, and 1,122 cm^–1^, respectively.

The FTIR spectrum of PEOM is also shown in Figure 2. By comparing the GE and PEO spectra, an interaction signal between PEO and GE was identified. The PEOM spectrum showed methylene, CH_2_ bending, −CH = CH- C bending, and O-C bending signals at 2,961, 1,383, 1,122, and 1,074 cm^–1^, also present in PEO. However, characteristic peaks at 3,434, 1,352, and 1,605 cm^–1^ belonging to GE were also found in the PEOM spectrum, indicating that PEO was successfully encapsulated with GE. The FTIR also suggests that PEO combines with GE by van der Waals forces, consistent with a report by Li et al. (43).

The structure was evaluated by XRD, and diffraction data are shown in Supplementary Figure 4. Both GE and PEOM showed broad peaks, indicating a structure of low crystallinity (44). GE presented the typical broad peak in the range of 10–30° (2θ), consistent with an earlier report (45). The XRD diffraction of PEOM was left-shifted compared to GE, demonstrating that PEOM was prepared successfully (46).

### 3.2. PEOM Thermal properties

#### 3.2.1. PEOM thermal stability

**FIGURE 3:**
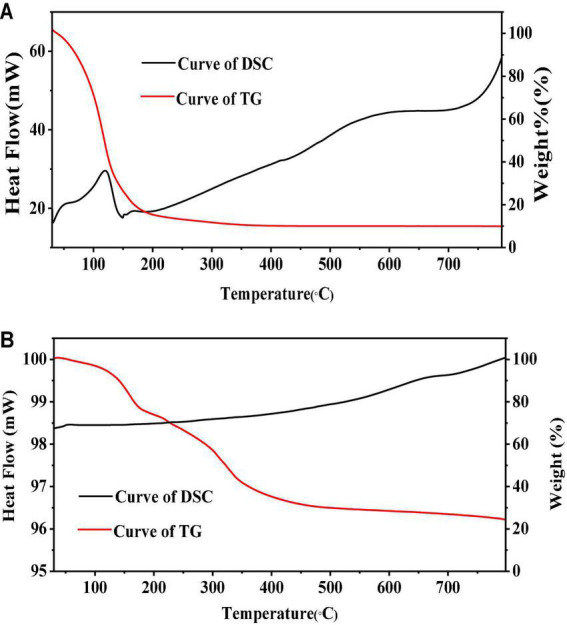
Thermogravimetric/differential scanning calorimetry (TG/DSC) of *Perilla* essential oil (PEO) and *Perilla* essential oil microcapsules (PEOM) [PEO **(A)** and PEOM **(B)**].

Thermogravimetry and DSC are effective ways to illustrate the stabilities and formation of microcapsules (47–49) by probing the relationship between temperature and mass variation (50). Results are shown in Figure 3. In a comparison of PEO and PEOM stabilities, Figure 3A shows the PEO mass variation with temperature. Both TG and DSC showed a mass loss up to 88.3% before 204°C. The temperature of maximum weight loss rate (0.025%/s) was 119°C, probably due to the volatilization of PEO. Meanwhile, the TG/DSC curves for PEOM are shown in Figure 3B. Comparing the two, PEO shows a single mass loss step, while PEOM presents two steps. A mass loss of about 3.3% before 102°C was found, attributed to the evaporation of water and PEO remaining on the surface of PEOM. In the 102–400°C range, TG/DSC showed a mass loss up to 76.3% from the volatilization or decomposition of PEOM. Comparison of the TG/DSC results for PEO and PEOM reveals that microencapsulation by simple coacervation effectively prevents the volatilization of PEO and confers excellent thermal stability. It is speculated that the release rate of PEO could be controlled by adjusting the temperature.

#### 3.2.2. Thermolysis of PEOM

To investigate changes in the chemical composition of PEO at high temperatures, thermolysis was studied using OLT-GC/MS. According to the TG/DSC results, PEOM thermolysis test temperatures were set to 100, 200, 300, and 400°C. Thermal decomposition material was collected in an offline pyrolysis tank (OLT) made by our group. The components of PEOM decomposition were analyzed by GC/MS, Supplementary Figure 5, and quantitative analysis was carried out using area percentages. Content with similarity over 90% is shown in Table 1.

**TABLE 1:** Thermal decomposition product of *Perilla* essential oil microcapsules (PEOM) at different temperature.

Time/min	Components	Similarity/%	100°C	200°C	300°C	400°C
8.387	Pinene	91	5.14 ± 0.07	0.98 ± 0.01	0.78 ± 0.03	0.58 ± 0.01
8.857	Cyclohexanol	95	21.35 ± 0.13	2.35 ± 0.07	3.56 ± 0.12	2.75 ± 0.09
8.874	Methylvaleric acid	91	19.86 ± 0.18	1.98 ± 0.09	3.05 ± 0.05	2.50 ± 0.09
10.644	Benzaldehyde	91	1.79 ± 0.03	2.05 ± 0.05	1.88 ± 0.03	1.64 ± 0.05
14.940	Benzeneethanol	95	/	0.58 ± 0.02	0.87 ± 0.05	0.69 ± 0.01
15.349	4-Isopropenyl-1-methylcyclohexene	94	/	1.84 ± 0.08	0.68 ± 0.04	1.03 ± 0.03
15.807	1-(3-Furyl)-4-methyl-1-pentanone	90	0.89 ± 0.03	0.98 ± 0.07	1.85 ± 0.11	3.65 ± 0.08
17.409	Nonyl aldehyde	97	/	0.98 ± 0.07	/	/
21.381	α-farnesene	91	1.05 ± 0.02	1.01 ± 0.08	0.85 ± 0.03	2.87 ± 0.12
21.752	1-caryophyllene	91	/	1.89 ± 0.08	2.98 ± 0.07	4.58 ± 0.05
21.794	trans-8-*p*-Menthen-7-ol	96	/	2.65 ± 0.15	1.82 ± 0.09	6.58 ± 0.17
23.404	5,6-dihydroxy-7-isopropyl-1,1-dimthyl-2,3,9,10-tetrahydrophenanthren-4-one	90	/	2.56 ± 0.15	3.58 ± 0.21	5.65 ± 0.17
23.425	3-(4-Methyl-3-pentenyl)-furan	93	4.56 ± 0.24	6.88 ± 0.28	7.59 ± 0.14	8.95 ± 0.17
24.125	(4*S*)-*p*-Mentha-1,8-dien-7-al	95	/	45.35 ± 0.34	53.81 ± 0.29	3.25 ± 0.11
25.631	2-methyl-2-butenal	91	/	3.56 ± 0.14	4.80 ± 0.28	1.28 ± 0.09
26.241	cis-3-hexenyl benzoate	90	/	0.85 ± 0.05	0.65 ± 0.02	2.45 ± 0.10
26.381	Pentadecane,	90	0.49	0.78 ± 0.05	1.18 ± 0.07	1.97 ± 0.09
35.864	Methyl hexadecanoate	90	0.34	7.16 ± 0.28	/	0.67 ± 0.15
28.408	Tetramethylpentadecane	92	/	1.65 ± 0.20	2.01 ± 0.09	3.56 ± 0.22
28.589	Triacontane	90	/	1.32 ± 0.17	1.87 ± 0.14	4.02 ± 0.09
29.108	Tetradecanoic acid	90	/	2.87 ± 0.05	3.58 ± 0.27	0.15 ± 0.08
29.858	Pentadecanoic acid	95	/	0.85 ± 0.05	0.74 ± 0.03	0.10 ± 0.01
30.047	Adamantyl Methacrylate	93	/	1.12 ± 0.08	1.25 ± 0.04	1.56 ± 0.08
40.998	Hexadecanoic acid	95	/	/	/	4.58 ± 0.41

As shown in Table 1, the main components of PEO are (4*S*)-*p-*mentha-1,8-dien-7-al, 3-(4-methyl-3-pentenyl)-furan, *trans*-8-*p*-menthen-7-ol, α-farnesene, methylvaleric acid, benzaldehyde, tetradecanoic acid, and 2-methyl-2-butenal. These results are consistent with reports by Ahmed et al. (51) and Wang et al. (52). At different temperatures, the main components released from PEOM were the same but at different concentrations. For example, the concentration of the main component [(4*S*)-*p*-mentha-1,8-dien-7-al] was 45.35, 53.81, and 3.25% at 200, 300, and 400°C, respectively, indicating that the optimal releasing temperature was in the range of 200–400°C, a result consistent with TG/DSC. Thermal decomposition analysis of PEOM demonstrates that encapsulation of PEO could effectively widen the scope of heat release applications.

### 3.3. *In vitro* release of PEOM

**FIGURE 4:**
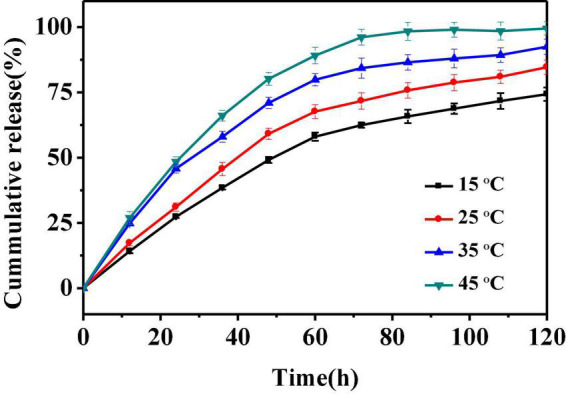
Release curves of *Perilla* essential oil microcapsules (PEOM) at different temperatures.

The above results confirm the successful preparation of PEOM with excellent thermal stability and provide a basis for development of PEOM delivery and preservation. To elucidate the storage properties, *in vitro* release was employed. The sustained release performance of PEOM was investigated by cumulative release at different temperatures, and zero order, first order, and Ritger-Peppas kinetics models were used to analyze the release mechanism (53). The release performance of PEOM was studied at 15, 25, 35, and 45°C for 120 h, and results were plotted in terms of cumulative release and time. Figure 4 shows that PEOM sustained PEO release with varying temperature. After 120 h, the cumulative release of PEOM at temperatures of 15, 25, 35, and 45°C was 74.34, 83.92, 92.47, and 99.52%, respectively. Two distinct stages were identified. Before 72 h, cumulative release increased substantially, stabilizing after 72 h. The result is consistent with that found by Sharkawy et al. (54) and may represent the rapid release of low boiling point and small molecules adsorbed on the surface of PEOM, and the gradual release of high boiling point and macromolecules from inside the PEOM which maintains the stability of the release rate (35). Comparison of the release curves at different temperatures indicates that the higher the temperature, the faster the release. The cumulative release of PEOM was 74.31% (15°C), 84.65% (25°C), 92.5% (35°C), and 99.5% (45°C) over 120 h. Reasons for this observation could be the increasing kinetic energy of PEO in PEOM. In addition, GE damaged by the increase in temperature would lead to larger pore size, decreased escape resistance, and increased permeability (55). These results demonstrate that adjusting the ambient temperature is an effective way to control cumulative release. Three kinetics models, namely the zero order, first order, and Ritger-Peppas models, were chosen to clarify the releasing mechanism of PEOM. These models are represented as follows:

Q=Kt⁢(zero⁢order)Q=1-exp⁢(-Kt)⁢(first⁢order)Q=Kt(Ritger-Peppas)n

where Q, K, t, and n are the cumulative release, constant, time, and the diffusion index, respectively.

The most optimal model for the release performance generally has a higher correlation coefficient (*R*^2^) (56). Table 2 and Supplementary Figures 6–8 show that the correlation coefficient of the first order model is greater than that of the other models (R^2^_15^°*C*_ = 0.9783; R^2^_25^°*C*_ = 0.9846; R^2^_35^°*C*_ = 0.9750; R^2^_45^°*C*_ = 0.9636). Therefore, the first order model is the most optimal for PEOM, best fitting the PEO release behavior in air atmosphere. The results indicate that *in vitro* release is an unstable process, that PEO was primarily released by diffusion from the wall material, the density of the wall causing diffusion resistance. These results are consistent with those of Dong et al. (57). In conclusion, PEOM can be stored at room temperature and has potential value for the preservation of food.

**TABLE 2:** Release kinetics of *Perilla* essential oil microcapsules (PEOM) at different ambient temperatures.

Kinetic models		15°C	25°C	35°C	45°C
Zero-order Q = Kt + C	*K*	0.6014	0.6772	0.6950	0.7699
	*C*	12.11	15.06	23.75	26.74
	*R* ^2^	0.9030	0.8843	0.9106	0.7862
First-order ln(100-Q) = Kt + C	*K*	-0.0116	-0.0157	-0.0213	-0.0467
	*C*	-0.0653	-0.0524	-0.1205	0.3118
	*R* ^2^	0.9783	0.9846	0.9750	0.9636
Rigter-Peppas Q = Kt^n^	lnK	1.0237	1.2704	2.0109	2.0667
	*n*	0.7124	0.6882	0.5512	0.5634
	*R* ^2^	0.9634	0.9562	0.9307	0.9154

### 3.4. PEOM antioxidant and antibacterial activity assay

#### 3.4.1. Antioxidant activity assay

**FIGURE 5:**
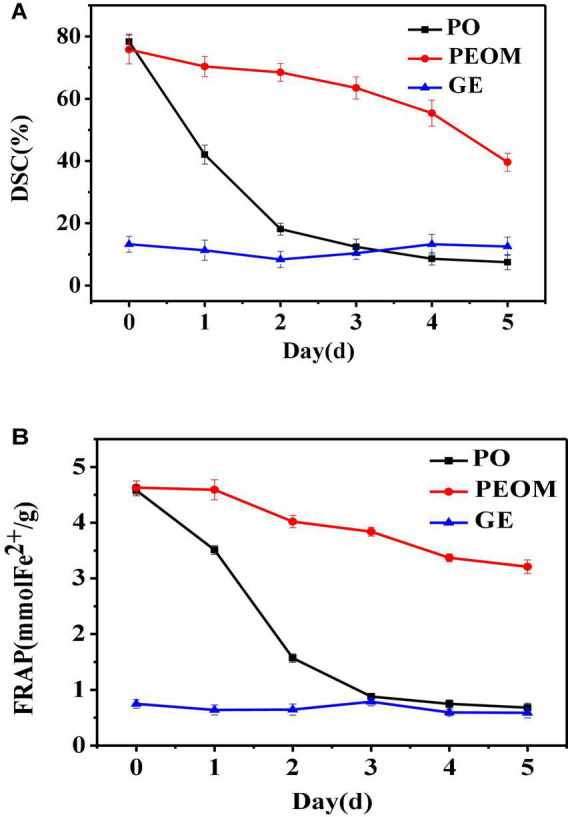
Antioxidant of *Perilla* essential oil (PEO) and *Perilla* essential oil microcapsules (PEOM) [DPPH **(A)** and FRAP **(B)**].

Reactive oxygen species and free radicals could lead to organic spoilage through lipid peroxidation and protein denaturation (58). The antioxidant activity of PEO encapsulated by GE could be more stable over time. To evaluate the antioxidant activities of PEOM and PEO during 5 days of storage, two antioxidation models were applied: 2,2-diphenyl-1-picrylhydrazyl (DPPH) scavenging capacity and FRAP. DPPH radical is widely used to study the scavenging activities of natural substances, and the FRAP method is applied to evaluate total antioxidant capacity by measuring the ability to reduce ferric iron to ferrous. Figure 5 shows the variation in antioxidant power of PEO, PEOM, and GE over 5 days. PEO and PEOM showed the most antioxidant potential at the beginning of the DPPH and FRAP experiments. As storage time increases, the antioxidant activity of PEOM weakened the slowest, while that of PEO weakened the more rapidly. At the end of the experiment, DPPH/FRAP of PEOM exhibited higher than that of PEO. The most probable reason was fast evaporation of PEO, followed a massive loss, leading to weaken rapidly in antioxidant capacity. Conversely, PEO in the PEOM must be penetrated the wall of GE, and gradual release under the same conditions. The results of comparison showed that the antioxidant activity of PEOM was found to be more sustainable than PEO, results consistent with those of Wang et al. (32).

#### 3.4.2. Antibacterial activity assay

**FIGURE 6:**
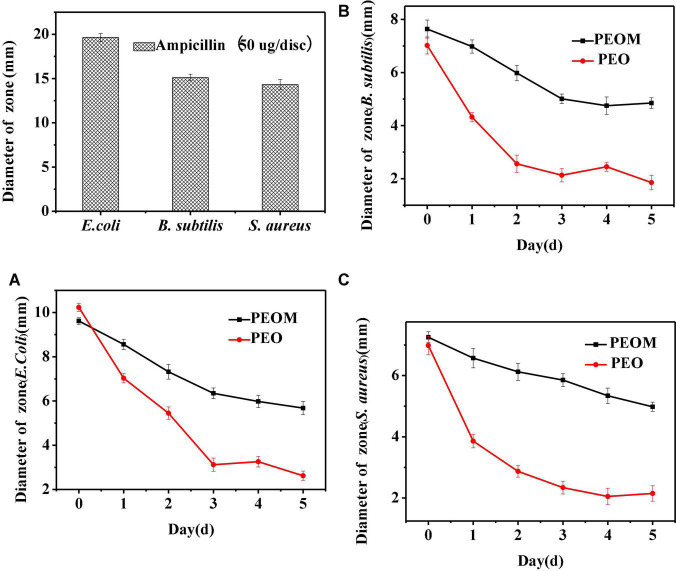
Antibacterial activity of *Perilla* essential oil (PEO), *Perilla* essential oil microcapsules (PEOM) and ampicillin. Diameter of zone of *E.coli* **(A)**, *B. subtilis* **(B)**, and *S. aureus* **(C)**.

Microbial proliferation is a key factor in organic spoilage. Although PEO has excellent antibacterial activity, PEOM could be more effective owing to the sustained release effect. In this paper, antibacterial activity of PEOM was investigated against *S. aureus*, *E. coli*, and *B. subtilis*. Figure 6 shows the variation in PEO and PEOM activity over 5 days. In the initial stages, antibacterial activities of PEO and PEOM were high for the three bacteria. Compared with PEO, the activity in PEOM fell slowly with increasing storage time demonstrating that encapsulation effectively maintains antibacterial activity, presumably by reducing the release rate. As a positive control, ampicillin showed excellent activity of 19.65 mM (*E. coli*), 15.12 mM (*B. subtilis*), and 14.32 mM (*S. aureus*) at a dose of 50 μg/dis. Jo et al. (59) found that stretch-responsive microcapsules could regulate the release of antibacterial compounds, results consistent with these findings.

### 3.5. Preservation assay

During their growth and reproduction processes, microorganisms produce a variety of enzymes which can destroy the cell wall, enter the cell interior and cause the decomposition of nutrients in foods (60). A variety of chemical bacteriostatic agents are applied in food preservation. However, chemical agents can be detrimental to human health and the environment (8). As a safe food additive, PEO shows excellent antioxidant and antimicrobial activities which have been applied to keep fruits fresh (61). Significantly, the PEO-loaded microcapsule PEOM prepared in this work exhibits stability and a sustained release effect, conferring stable antioxidant and antibacterial activities. Therefore, PEOM was investigated in peach preservation, evaluating change in appearance, hardness, TA, SSC, and total bacterial counts of peach fruit.

After fruit picking, disorderly change of organisms occurs due to respiration, carbohydrate metabolism, and enzymatic degradation. Change in appearance is an important factor to indicate the freshness of the fruit. Untreated and treated peaches during storage are shown in Supplementary Figure 9. After 3 days, the PEOM treated fruit exhibited no rotting or evaporation of water, while untreated fruit were starting to go rotten. Partial collapse and a rotten core were found in the untreated group after 9 days. However, the treated group only showed a small amount of mucedine after 9 days of storage, indicating that PEOM can effectively prolong the shelf-life of peach at room temperature. In the initial stages, the preservation effect was not obvious for the treated group, perhaps due to a low PEO concentration. However, with the extended release of PEO in the middle and late stages, the growth and proliferation of bacteria were effectively prevented, resulting in preservation of the fruit. These findings agree with the report by Siroli et al. (62) on apple preservation with citral microcapsules.

Due to pectin action, the cell of the fruit is decomposed during storage, leading to tenderizing (63). Initially, protopectin decomposes into pectin, which is converted into pectin acid, causing cell decomposition and loss of hardness. Table 3 shows the hardness of peaches during storage, the hardness of treated and untreated groups decreasing gradually. However, the treated group exhibited a lower rate of decrease due to the sustained release of PEOM and inhibition of the decomposition rate of protopectin. Maintaining the hardness of the peaches, PEOM demonstrates a positive effect on their preservation.

**TABLE 3:** *Perilla* essential oil microcapsules (PEOM) on preservation of peach.

		0 day	2 days	4 days	6 days	8 days	10 days
Hardness (g)	Tg	1,654 ± 20	1,258 ± 23	1,063 ± 28	1,058 ± 25	948 ± 24	845 ± 22
	Utg	1,605 ± 19	1,425 ± 25	1,015 ± 17	845 ± 25	732 ± 21	503 ± 19
SSC (%)	Tg	10.7 ± 0.2	10.2 ± 0.1	10.4 ± 0.3	9.5 ± 0.2	9.3 ± 0.2	8.9 ± 0.3
	Utg	10.3 ± 0.4	9.8 ± 0.3	9.3 ± 0.4	7.5 ± 0.3	6.9 ± 0.4	6.1 ± 0.1
TA (mg/g)	Tg	8.82 ± 0.13	8.51 ± 0.07	7.93 ± 0.11	7.65 ± 0.07	7.38 ± 0.15	7.09 ± 0.13
	Utg	9.05 ± 0.15	9.32 ± 0.12	7.84 ± 0.07	6.72 ± 0.06	6.50 ± 0.10	6.34 ± 0.09
TBC (CFU/g)	Tg	1.71 ± 0.15	2.24 ± 0.21	2.56 ± 0.18	3.02 ± 0.34	3.31 ± 0.24	4.02 ± 0.18
	Utg	1.63 ± 0.21	2.32 ± 0.15	3.56 ± 0.10	4.15 ± 0.08	5.58 ± 0.11	6.78 ± 0.23

TG is treated group. UTG is untreated group.

Soluble solid content is a measure of sweetness, whereby the metabolism and respiration of fruit decrease sugar, vitamin, and mineral content (64). Therefore, SSC was measured during storage, identifying a gradual decrease, Table 3. The treated group mass was higher than that of the untreated group after 4 days. Due to the sustained release of PEO and the controlled inhibition of respiration intensity by PEOM, conversion of nutrients was slowed down and the SSC of the treated group was higher than that of the control group.

The growth rate of bacteria increases with acid conversion by respiratory action, resulting in accelerated rotting of fruit (7). As an important quality parameter, the TA of peach was evaluated in this study, finding a decrease in TA content with time for all the groups. After 4 days, the TA for the treated groups was higher than for the untreated groups. Sustained release of PEO from PEOM inhibits respiration and reduces acid conversion before 4 days, causing the TA of the treated group to be higher than that of the control groups. However, TA increased slightly after 4 days, perhaps due to lactic acid from respiration. These hardness, TA, and SSC results agree with earlier findings on preservation with essential oil-loaded microcapsules (35, 65).

According to the Duncan test, values in the same line are not significantly different (*P* > 0.05).

Microbial growth on fruit carbohydrates results in spoilage (66). Therefore, TBC are an important measure of the quality of fresh fruits. If the safe edible value of fruit is over 6 log CFU/g, it may not be safe to eat (67). TBC for peach for both treated and untreated groups are shown in Table 3. TBC of the treated groups increased from 2.56 ± 0.18 to 4.02 ± 0.18 log CFU/g after 4 days, a small increase due to bacterial growth inhibition by PEO sustained release. In contrast, the untreated group after 4 days experienced a rapid increase of TBC from 3.56 ± 0.10 to 6.78 ± 0.23 log CFU/g, over the safely edible value limit by the 10 day. This could be attributed to an increase in microbial respiration. The results demonstrate that PEOM shows an excellent antibacterial effect in the preservation of fresh peaches, reducing the volatility and controlling the release rate of PEO with long-lasting effects. Concurrent and consistent with this study, Li et al. (68) have described that *P. frutescens* essential oil can delay the rot of strawberries.

### 3.6. Cost analysis

Cold storage preservation was the most frequently applied in fresh-cut fruit, which was used to compare with PEOM method, and cost analysis was evaluated. As shown in Supplementary Table 1, it was clearly demonstrated the cost of PEOM was lower than the cold storage preservation for electric bill and investment of refrigerator.

In summary, PEO-loaded microcapsules exhibited excellent stability, sustained release effects, and effectively maintained PEO antioxidant and antibacterial activities, resulting in an extended peach shelf-life.

## 4. Conclusion

In this paper, temperature-controlled PEOM was developed to decrease the volatility and increase the stability of PEO for the preservation of fresh peaches. PEOM prepared with a core-shell ratio of 1.4:1 exhibited excellent encapsulation efficiency (91.5%) and a smoother microcapsule surface. PEOM showed excellent stability and the PEO release rate could be controlled with temperature. Due to its temperature-responsive character, *in vitro* release behavior proved that the optimal kinetics model of PEOM fitted well with first order. The PEO release mechanism primarily involves diffusion from the wall material, with diffusion resistance from the wall density. The loss of PEO is reduced and the rate of release is sustained, maintaining the antioxidant and antibacterial activities of PEOM at a higher level during storage. And, its cost was lower than the storage preservation for electric bill and investment of refrigerator. Therefore, preservation with PEOM could protect the quality and prolong the shelf-life of peaches. The above findings are promising for PEOM applications as preservatives in the food industry.

## Data availability statement

The original contributions presented in this study are included in this article/Supplementary material, further inquiries can be directed to the corresponding authors.

## Author contributions

ZT and ChuX: conceptualization. MZ: methodology, formal analysis, data curation, and writing—review and editing. ZL: validation and funding acquisition. ZT: investigation, writing—original draft preparation, and supervision. ChuX: resources and project administration. All authors have read and agreed to the published version of the manuscript.

## References

[1] Doets Esmée L Kremer S. The silver sensory experience – A review of senior consumers’ food perception, liking and intake. *Food Qual Prefer.* (2016) 48:316–32. 10.1016/j.foodqual.2015.08.010

[2] Ju J Chen XQ Xie YF Yu H Guo YH Cheng YL Application of essential oil as a sustained release preparation in food packaging. *Trends Food Sci Technol.* (2019) 92:22–32. 10.1016/j.tifs.2019.08.005

[3] Ju J Xie YF Guo YH Cheng YL Qian H Yao WR. Application of starch microcapsules containing essential oil in food preservation. *Crit Rev Food Sci.* (2020) 60:2825–36. 10.1080/10408398.2018.1503590 30040433

[4] Phromnoi K Suttajit M Saenjum C Dejkriengkraikul PL. Inhibitory effect of a rosmarinic acid-enriched fraction prepared from Nga-Mon (*Perilla frutescens*) seed meal on osteoclastogenesis through the RANK signaling pathway. *Antioxidants.* (2021) 10:307. 10.3390/antiox10020307 33671207 PMC7923133

[5] Hu ZY Lu C Zhang YS Tong WJ Du LH Liu F. Proteomic analysis of *Aspergillus flavus* reveals the antifungal action of *Perilla frutescens* essential oil by interfering with energy metabolism and defense function. *LWT Food Sci Technol.* (2021) 154:112660. 10.1016/j.lwt.2021.112660

[6] Chen BQ. Edible spice plants in xishuangbanna ethnic minority. *Flavour Fragr Cosmet.* (1991) 1:27–31. 10.1186/s13002-017-0158-7 28506271 PMC5433000

[7] Li N Zhang ZJ Li XJ Li HZ Cui LX He DL. Microcapsules biologically prepared using *Perilla frutescens* (l.) Britt. essential oil and their use for extension of fruit shelf life. *J Sci Food Agric.* (2017) 98:1033. 10.1002/jsfa.8552 28718920

[8] Chen Z Wu K Zhu W Wang Y Su C Yi F. Chemical compositions and bioactivities of essential oil from *Perilla* leaf (perillae folium) obtained by ultrasonic-assisted hydro-distillation with natural deep eutectic solvents. *Food Chem.* (2022) 375:131834. 10.1016/j.foodchem.2021.131834 34920308

[9] Chen FL Liu SS Zhao ZY Gao WB Ma YB Wang XX Ultrasound pre-treatment combined with microwave-assisted hydrodistillation of essential oils from *Perilla frutescens* (L.) Britt. leaves and its chemical composition and biological activity. *Ind Crop Prod.* (2020) 143:111908. 10.1016/j.indcrop.2019.111908

[10] Hu ZY Yuan K Zhou Q Lu C Du LH Liu F. Mechanism of antifungal activity of *Perilla frutescens* essential oil against *Aspergillus flavus* by transcriptomic analysis. *Food Control.* (2021) 123:107703. 10.1016/j.foodcont.2020.107703

[11] Cimino C Maurel OM Musumeci T Bonaccorso A Drago F Souto EMB Essential oils: pharmaceutical applications and encapsulation strategies into lipid-based delivery systems. *Pharmaceutics.* (2021) 13:327. 10.3390/pharmaceutics13030327 33802570 PMC8001530

[12] Xie WC Gu XH Tan ZC Tang J Wang GY Luo CR Thermal decomposition of two synthetic glycosides by tg, dsc and simultaneous py-gc-ms analysis. *J Therm Anal Calorim.* (2007) 87:505–10. 10.1007/s10973-006-7617-z

[13] Woźniak Ł Marszałek K Skąpska S. Influence of steviol glycosides on the stability of vitamin C and anthocyanins. *J Agric Food Chem.* (2014) 62:11264–9. 10.1021/jf504001t 25376304

[14] Yang K Liu AP Hu AX Li JX Zen Z Liu YT Preparation and characterization of cinnamon essential oil nanocapsules and comparison of volatile components and antibacterial ability of cinnamon essential oil before and after encapsulation. *Food Control.* (2021) 123:107783. 10.1016/j.foodcont.2020.107783

[15] Sun YN Zhang M Bhandari B Bai B. Fennel essential oil loaded porous starch-based micro-encapsulation as an efficient delivery system for the quality improvement of ground pork. *Int J Biol Macromol.* (2021) 172:464–74. 10.1016/j.ijbiomac.2021.01.090 33465361

[16] Ping S Jiang Y Chen HJ Gao HY. Development of microcapsule bioactive paper loaded with cinnamon essential oil to improve the quality of edible fungi. *Food Packaging Shelf.* (2021) 27:100617. 10.1016/j.fpsl.2020.100617

[17] Chatterjee S Judeh Z. Encapsulation of fish oil with n-stearoyl o-butylglyceryl chitosan using membrane and ultrasonic emulsification processes. *Carbohydr Polym.* (2015) 123:432–42. 10.1016/j.carbpol.2015.01.072 25843877

[18] Ren WB Tian GF Zhao SJ Yang Y Gao W Zhao CY Effects of spray-drying tem-perature on the physicochemical properties and polymethoxyflavone loading efficiency of citrus oil microcapsules. *LWT Food Sci Technol.* (2020) 133:109954. 10.1016/j.lwt.2020.109954

[19] Lee H Choi CH Abbaspourrad A Wesner C Caggioni M Zhu TT Encapsulation and enhanced retention of fragrance in polymer microcapsules. *ACS Appl Mater Inter.* (2016) 8:4007–13. 10.1021/acsami.5b11351 26799189

[20] Bertrand M Wang XJ Xia SQ Zhang XM Li Y Zhang S. Microencapsulation of essential oils by complex coacervation method: preparation, thermal stability, release properties and applications. *Crit Rev Food Sci.* (2020) 62:1363–82. 10.1080/10408398.2020.1843132 33176432

[21] Lazko J Popineau Y Legrand J. Soy glycine microcapsules by simple coacervation method. *Colloids Surf B Biointerfaces.* (2004) 37:1–8. 10.1016/j.colsurfb.2004.06.004 15450301

[22] Assadpour E Jafari SM. Advances in spray-drying encapsulation of food bioactive ingredients: from microcapsules to nanocapsules. *Annu Rev Food Sci Technol.* (2019) 10:103–31. 10.1146/annurev-food-032818-121641 30649963

[23] Martins IM Barreiro MF Coelho M Rodrigues AE. Microencapsulation of essential oils with biodegradable polymeric carriers for cosmetic applications. *Chem Eng J.* (2014) 245:191–200. 10.1016/j.cis.2021.102544 34717207

[24] Chen L Li R Zhang LJ Jiang ZT. Preparation and performance studies on the microencapsule of perilla oil entrapped with complex coacervation. *Sci Technol Food Ind.* (2015) 3:232–8.

[25] Zhao WW Wang YL. Coacervation with surfactants: from single-chain surfactants to gemini surfactants. *Adv Colloid Interface.* (2017) 239:199–212. 10.1016/j.cis.2016.04.005 27260407

[26] Wu KG Xiao Q. Microencapsulation of fish oil by simple coacervation of hydroxypropyl methyl-cellulose. *Chin J Chem.* (2005) 23:1569–72.

[27] Gu XL Zhu X Kong XZ Tan YB. Comparisons of simple and complex coacervations for preparation of sprayable insect sex pheromone microcapsules and release control of the encapsulated pheromone molecule. *J Microencapsul.* (2010) 27:355–64. 10.3109/02652040903221532 20163286

[28] Chen MJ Hu Y Zhou J Xie YR Wu H Yuan T Facile fabrication of tea tree oil-loaded antibacterial microcapsules by complex coacervation of sodium alginate/quaternary ammonium salt of chitosan. *RSC Adv.* (2016) 6:13032–9. 10.1039/C5RA26052C

[29] Jiang AY Ma HJ Song GB Zhang FM Liu ZH Li ZJ The preparation and heat release of sweet orange oil microcapsule. *J Yunnan Univ Nat Sci Edition.* (2021) 43:753–9.

[30] Xiong D Yin TZ Xie C Ma HJ Song GB Cai YJ Preparation of inclusion complex of cyclodextrin with menthyl-D-glycoside and research on its heat release properties. *Chem Res Appl.* (2022) 34:1501–8.

[31] Li ZJ Liu ZH Tai ZG Jiang KM He P Xiang NJ *Thermal Decomposition Tank and Method for Decomposing and Collecting Sustained-Release Perfume Ingredients.* Patent: CN112858541 A. (2021).

[32] Wang HH Li MY Dong ZY Zhang TH Yu QY. Preparation and characterization of ginger essential oil microcapsule composite films. *Foods.* (2021) 10:2268. 10.3390/foods10102268 34681317 PMC8534594

[33] Tai ZG Cai L Dai L Dong L Wang M Yang Y Antioxidant activity and chemical constituents of edible flower of *Sophora viciifolia*. *Food Chem.* (2011) 126:1648–54. 10.1016/j.foodchem.2010.12.048 25213940

[34] Cristina Gomes Da S Eliana Della Coletta Y Priscila Almeida Lucio C Derval Dos Santos R. The performance evaluation of Eugenol and Linalool microencapsulated by PLA on their activities against pathogenic bacteria. *Mater Today Chem.* (2021) 21:100493.

[35] Ban ZJ Zhang JL Li L Luo ZS Wang YJ Yuan QP. Ginger essential oil-based microen-capsulation as an efficient delivery system for the improvement of Jujube (*Ziziphus jujuba* Mill.) fruit quality. *Food Chem.* (2020) 306:125628. 10.1016/j.foodchem.2019.125628 31629297

[36] Qiang G Peng W Tong N He DJ Wang ML Yang HJ Soluble solid content and firmness index assessment and maturity discrimination of *Malus micromalus* Makino based on near-infrared hyperspectral imaging. *Food Chem.* (2021) 370:131013. 10.1016/j.foodchem.2021.131013 34509150

[37] Yin Q Guo ML Li W Ren HZ Wang JP Zhang XX. Fabrication and performance of pol-yurethane/polyurea microencapsulated phase change materials with isophorone diisocyanate via interfacial polymerization. *Sci Adv Mater.* (2019) 11:647–54.

[38] Cao LH Luo J Tu KH Wang LQ Jiang HL. Generation of nano-sized core-shell particles using a coaxial tri-capillary electrospray-template removal method. *Colloid Surface B Biointerfaces.* (2014) 115:212–8. 10.1016/j.colsurfb.2013.11.046 24362060

[39] Li Q Hou L Wang H Zhang WY. Preparation of fish roe microcapsules by using micro-fluidics digitalization. *CIESC J.* (2011) 62:1042–7.

[40] Ocak B Gülümser G Baloğlu E. Microencapsulation of *Melaleuca alternifolia* (tea tree) oil by using simple coacervation method. *J Essent Oil Res.* (2011) 23:58–65.

[41] Tam SK Dusseault J Polizu S Ménard M Hallé JP Yahia L. Physicochemical model of alginate-poly-l-lysine microcapsules defined at the micrometric/nanometric scale using atr-ftir, xps, and tof-sims. *Biomaterials.* (2005) 26:6950–61. 10.1016/j.biomaterials.2005.05.007 15975648

[42] Hashim DM Man YBC Norakasha R Shuhaimi M Salmah Y Syahariza ZA. Potential use of fourier transform infrared spectroscopy for differentiation of bovine and porcine gelatins. *Food Chem.* (2010) 118:856–60. 10.5740/jaoacint.17-0244 28886757

[43] Li M Yu H Xie YF Guo YH Cheng YL Qian H Fabrication of eugenol loaded gelatin nanofibers by electrospinning technique as active packaging material. *LWT.* (2021) 139:110800. 10.1016/j.colsurfb.2022.112743 35961113

[44] Shu B Yu WL Zhao YP Liu XY. Study on microencapsulation of lycopene by spray-drying. *J Food Eng.* (2005) 76:664–9.

[45] Wan Y Zuo G Chao L Li X Luo H. Preparation and characterization of nano-platelet-like hydroxyapatite/gelatin nanocomposites. *Polym Adv Technol.* (2011) 22:2659–64.

[46] Yücetepe M Bayiit B Karaaslan M. Design of novel nutritious microcapsules comprising ω-5 fatty acids and essential amino acids by assembling pomegranate seed derived macromolecules. *LWT Food Sci Technol.* (2021) 143:111162. 10.1016/j.lwt.2021.111162

[47] Healy C Patil KM Wilson BH Hermanspahn L Harvey-Reid NC Howard BI. The thermal stability of metal-organic frameworks. *Coordin Chem Rev.* (2020) 419:213388. 10.1016/j.ccr.2020.213388

[48] Kroon MC Buijs W Peters CJ Witkamp GJ. Quantum chemical aided prediction of the thermal decomposition mechanisms and temperatures of ionic liquids. *Thermochimi Acta.* (2007) 465:40–7.

[49] Zheng, Li X Wei LX Dong LW. Study on development and permeability properties of thermal sensitive microcapsules for display material. *J Funct Mater.* (2012) 43:1813–6.

[50] de Souza SMM e Melo Franco PIB Leles MIG da Conceição EC. Evaluation of thermal stability of enalapril maleate tablets using thermogravimetry and differential scanning calorimetry. *J Therm Anal Calorim.* (2016) 123:1943–9.

[51] Ahmed HM Tavaszi-Sarosi S. Identification and quantification of essential oil content and composition, total polyphenols and antioxidant capacity of *Perilla frutescens* (L.) Britt. *Food Chem.* (2019) 275:730–8. 10.1016/j.foodchem.2018.09.155 30724256

[52] Wang J Xue S Zhao GH. Comparative study on chemical components and in vitro antioxidant capacity of essential oil from different parts of *Perilla frutescens*. *Food Sci.* (2013) 34:86–91.

[53] Teng XX Zhang M Mujumdard AS Wang HQ. Garlic essential oil microcapsules prepared using gallic acid grafted chitosan: effect on nitrite control of prepared vegetable dishes during storage. *Food Chem.* (2022) 388:132945. 10.1016/j.foodchem.2022.132945 35472626

[54] Sharkawy A Fernandes IP Barreiro MF Rodrigues AE Shoeib T. Aroma-loaded micro-capsules with antibacterial activity for eco-friendly textile application: synthesis, characterization, release, and green grafting. *Ind Eng Chem Res.* (2017) 56:5516–26.

[55] Hwang CM Sant S Masaeli M Kachouie NN Zamanian B Lee SH Fabrication of three-dimensional porous cell-laden hydrogel for tissue engineering. *Biofabrication.* (2010) 2:035003. 10.1088/1758-5082/2/3/035003 PMC3282162 20823504

[56] Tebcharani L Wanzke C Lutz TM Rodon-Fores J Lieleg O Boekhoven J. Emulsions of hydrolyzable oils for the zero-order release of hydrophobic drugs. *J Control Release.* (2021) 339:498–505. 10.1016/j.jconrel.2021.10.014 34662584

[57] Dong ZJ Wang Y Xia SQ Jia CS Zhang XM Xu SY. Release kinetics and application of spherical multinuclear peppermint oil microcapsules prepared by complex coacervation. *Food Sci.* (2010) 31:6–9.

[58] Shi C Knochel S. Inhibitory effects of binary combinations of microbial metabolites on the growth of tolerant *Penicillium roqueforti* and *Mucor circinelloides*. *LWT Food Sci Technol.* (2021) 149:112039. 10.1016/j.lwt.2021.112039

[59] Wang XD Ma H Guan CY Guan M. Germplasm screening of green manure rapeseed through the effects of short-term decomposition on soil nutrients and microorganisms. *Agriculture.* (2021) 11:1219.

[60] Saylan Y Akgönüllü S Denizli A. Plasmonic sensors for monitoring biological and chemical threat agents. *Biosensors.* (2020) 10:142. 10.3390/bios10100142 33076308 PMC7602421

[61] Yun KJ Heo SJ Peredo AP Mauck RL Dodge GR Lee D. Stretch-responsive adhesive microcapsules for strain-regulated antibiotic release from fabric wound dressings. *Biomater Sci UK.* (2021) 9:5136–43. 10.1039/d1bm00628b 34223592 PMC8320384

[62] Siroli L Patrignani F Serrazanetti DI Vannini L Salvetti E Torriani S Use of a nisin-producing *Lactococcus lactis* strain, combined with natural antimicrobials, to improve the safety and shelf-life of minimally processed sliced apples. *Food Microbiol.* (2016) 54:11–9.

[63] Xiao L Bi JF Xin J Xuan L Zhao YY Song Y. Effect of pectin osmosis or degradation on the water migration and texture properties of apple cube dried by instant controlled pressure drop drying (DIC). *LWT Food Sci Technol.* (2020) 125:109202.

[64] Jiang YL Yin H Wang DF Zhong Y Deng Y. Combination of chitosan coating and heat shock treatments to maintain postharvest quality and alleviate cracking of *Akebia trifoliate* fruit during cold storage. *Food Chem.* (2022) 394:133330. 10.1016/j.foodchem.2022.133330 35752120

[65] Sun Y Huang Y Wang XY Wu ZY Weng YX. Kinetic analysis of PGA/PBAT plastic films for strawberry fruit preservation quality and enzyme activity. *J Food Compost Anal.* (2022) 108:104439.

[66] Cai C Ma R Duan M Deng Y Lu D. Effect of starch film containing thyme essential oil mi-crocapsules on physicochemical activity of mango. *LWT Food Sci Technol.* (2020) 131:109700.

[67] Mewa-Ngongang M du Plessis HW Ntwampe SKO Chidi BS Hutchinson UF Mekuto L The use of candida pyralidae and *Pichia kluyveri* to control spoilage microorganisms of raw fruits used for beverage production. *Foods.* (2019) 8:454. 10.3390/foods8100454 31590435 PMC6835701

[68] Glicerina V Siroli L Betoret E Canali G Dalla Rosa M Lanciotti R Characterization and evaluation of the influence of an alginate, cocoa and a bilayer alginate-cocoa coating on the quality of fresh-cut oranges during storage. *J Sci Food Agric.* (2022) 102:4454–61. 10.1002/jsfa.11799 35092615

