# Supplementary material for: Temperature controlled microcapsule loaded with Perilla essential oil and its application in preservation of peaches
Source: Front Nutr. 2023 Feb 6;10:1087605. doi: 10.3389/fnut.2023.1087605 (PMC9939902; doi:10.3389/fnut.2023.1087605)
Supplement: Supplementary file 1 [file Data_Sheet_1.docx]

**Temperature controlling microcapsule loaded with perilla essential oil and its application in preservation of peach**

Zhigang Tai^1 *^, Minjie Zheng^1†^ , Ye Yang^1^, Cheng Xie ^1^, Zhenjie Li^2^, Chunpin Xu^3*^

^1^ Faculty of Life Science and Technology, Kunming University of Science and Technology, Kunming, 650500, P.R. China

^2^ Yunnan Key Laboratory of Tobacco Chemistr y, R&D Center of China Tobacco Yunnan Industry Co., Ltd., Kunming, 650231, P.R. China

^3^ College of Food and Bioengineering, Zhengzhou University of Light Industry, Zhengzhou, 450002, P.R. China


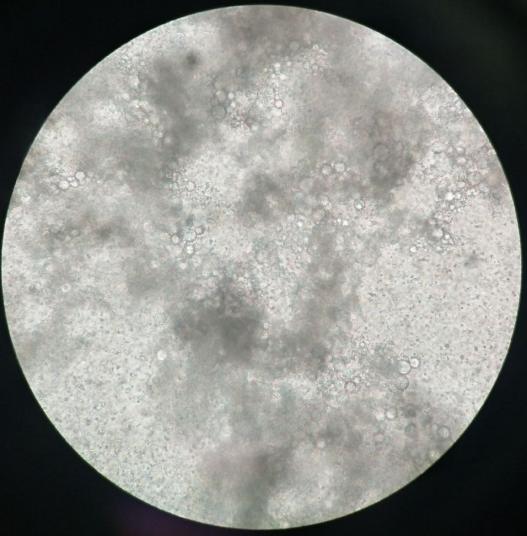
 **
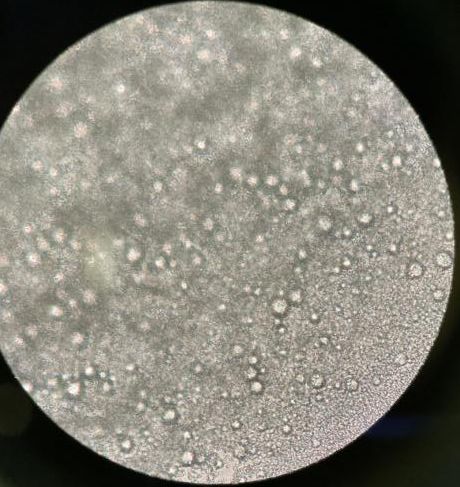
**

**Figure S1.** Inverted microscope of PEOM


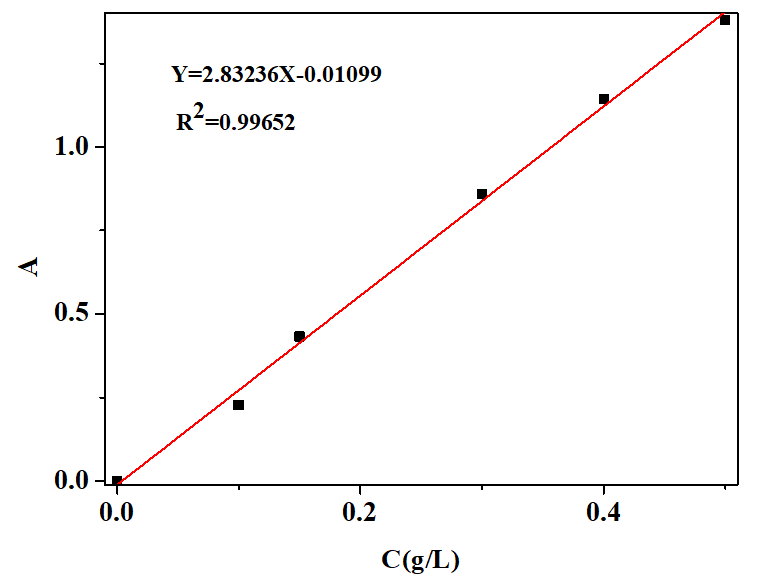


**Figure S2.** Calibration curve of absorbance versus PEO concentration

**Figure S3.** Size distribution of microstructure of PEOM

**Figure S4.** XRD spectroscopy of PEOM

**Figure S5.** Thermal decomposition of PEOM by using GC/MS

**Figure S6.** Zeroth order kinetic release of PEOM at different temperatures

**Figure S7.** First-order kinetic release of PEOM at different temperatures

**Figure S8.** Ritger-Peppas kinetic release of PEOM at different temperatures


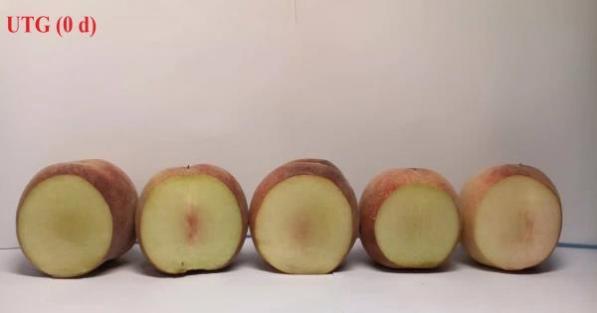

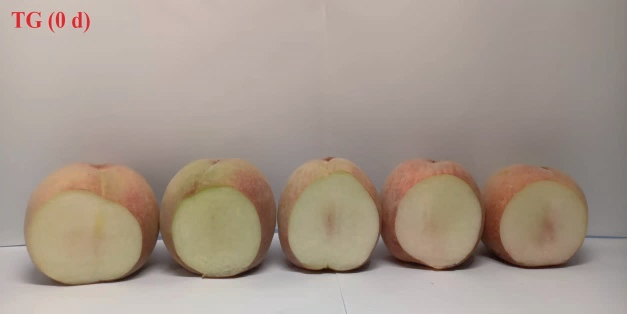


**
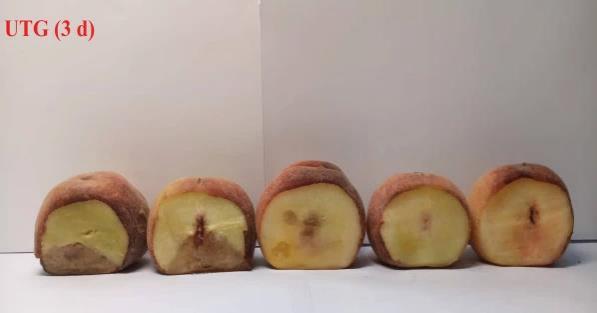

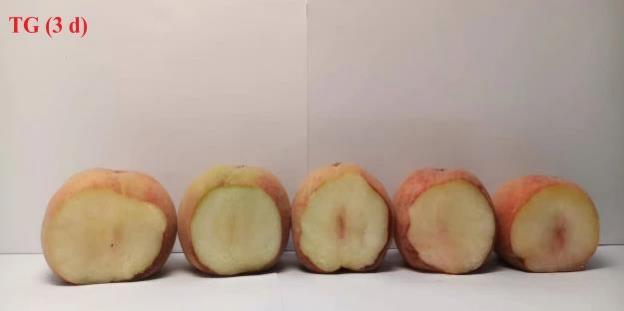
**

**
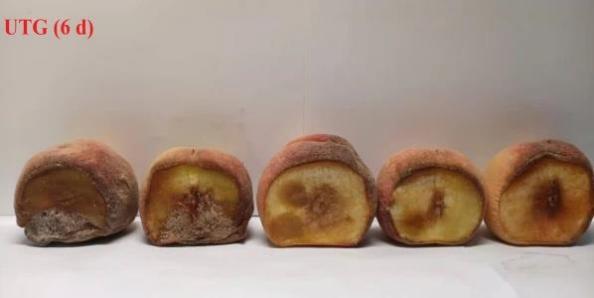

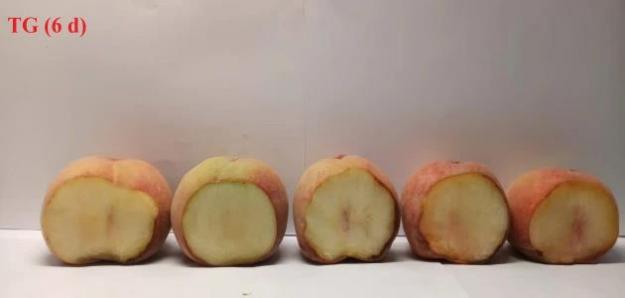
**

**
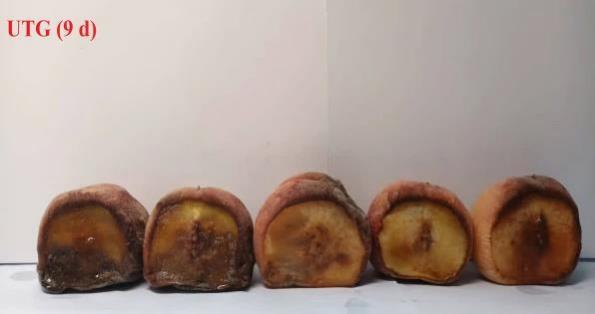

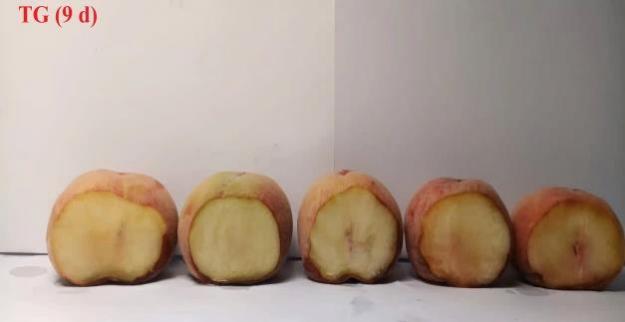
**

**Figure S9.** Changes appearance of peach during storage.[ TG is treated group, and UTG is untreated group]

**Table S1. Cost analysis of PEOM and cold storage preservation (10 Days).**

| Method | Details  0.65 g PEO/Kg peach) | Total  (Yuan) | Method | Details  (1.0 kg peach) | Total  (Yuan) | |
| --- | --- | --- | --- | --- | --- | --- |
| PEOM | PEOM(3.4 Yuan) | 8.0 | Cold Storage preservation | electric bill  (11.0 Yuan) | 461.0 Yuan | |
|  | Gelatin (0.60 Yuan) |  |  | Refrigerator (450Yuan) |  | |
|  | consumption of solvent (4.0 Yuan) |  |  |  |  |  |
|  | | | | | |  |
